# Better treatment outcomes in patients with actively treated therapy-related myeloid neoplasms harboring a normal karyotype

**DOI:** 10.1371/journal.pone.0209800

**Published:** 2018-12-31

**Authors:** Sang-A Kim, Junshik Hong, Woo Chan Park, Dong-Yeop Shin, Youngil Koh, Inho Kim, Dong Soon Lee, Sung-Soo Yoon

**Affiliations:** 1 Department of Internal Medicine, Seoul National University College of Medicine, Seoul National University Hospital, Seoul, Korea; 2 Cancer Research Institute, Seoul National University Hospital, Seoul, Korea; 3 Department of Laboratory Medicine, Seoul National University College of Medicine, Seoul National University Hospital, Seoul, Korea; University of Texas MD Anderson Cancer Center, UNITED STATES

## Abstract

We analyzed treatment outcomes and prognostic factors in adult patients with therapy-related myeloid neoplasms (t-MNs) to select patients who would be benefited by active anticancer treatment. After excluding 18 patients who received palliative care only and 13 patients with acute promyelocytic leukemia, 72 t-MN patients (45 with acute myeloid leukemia and 27 with myelodysplastic syndrome) were retrospectively evaluated. Among them, 10 (13.9%), 32 (44.4%), and 30 patients (41.7%) had favorable, intermediate- and adverse-risk cytogenetics, respectively. Among patients with intermediate-risk cytogenetics, patients with a normal karyotype (NK; N = 20) showed superior allogeneic stem cell transplantation-censored overall survival (AC-OS) and OS compared to those with non-NK-intermediate-risk cytogenetics (*P* < 0.001). In the multivariate analysis, male sex, age ≥ 70 years, and unfavorable cytogenetics (non-NK-intermediate plus adverse risk cytogenetics) were associated with inferior AC-OS. Those results suggest that a more-refined subdivision of risk stratification would be necessary in patients with intermediate-risk cytogenetics.

## Introduction

Therapy-related myeloid neoplasms (t-MNs) are myeloid malignancies diagnosed after previous exposure to cytotoxic agents employed for therapeutic purposes, mostly cytotoxic chemotherapy (CT) or ionizing radiation therapy (RT) for cancer treatment [1]. t-MNs include therapy-related myelodysplastic syndrome (t-MDS) and therapy-related acute myeloid leukemia (t-AML). t-MNs are one of the lethal long-term complications after anticancer CT/RT. Because almost every t-MDS eventually evolves to t-AML and similar therapeutic interventions are considered, these diseases are taken together as one distinct category in the 2016 World Health Organization (WHO) classification [2], and investigators often analyze them together [3, 4].

It is known that t-MNs have a worse prognosis than de novo MNs. Impaired organ function caused by toxicities of treatment for preceding cancer (PC) as well as biological resistance to CT/RT results in lower rates [5] and shorter durations [6] of complete remission (CR) after induction CT, leading to a 5-year overall survival (OS) rate of less than 10% [7]. However, the simple negative view of t-MNs is not always appropriate, for the following reasons: First, some t-MN patients with a favorable genetic risk category can achieve a good treatment outcome. A recent international study that evaluated 103 adult therapy-related acute promyelocytic leukemia (t-APL) patients in the U.S. and Europe reported that patients treated with arsenic trioxide-integrated therapy had a significantly better event-free survival (EFS) than those treated with intensive chemotherapy (IC) plus all-trans retinoic acid (ATRA; 95% vs. 78%; *P* = 0.042), and their 2-year OS rate was 88%, comparable to that of de novo APL patients [8]. Second, some patients with t-MNs may have a good performance status (PS) and be suitable for allogeneic hematopoietic stem cell transplantation (HSCT), for which the outcomes have significantly improved in recent years by the use of alternative donors, reduced intensity conditioning, and better infection prophylaxis [9]. Because previous retrospective studies included patients who could not tolerate active treatment and received best supportive care (BSC) only, the outcome of actively treated t-MN patients may be superior to the outcome (i.e., the reported OS) in the whole population. Therefore, a biased skepticism for all t-MN patients may result in the risk of undertreating patients who can otherwise be well cured.

The prognostic stratification of t-MN patients according to their pathogeneses and clinical characteristics is important for selecting patients who can be cured or at least significantly benefited by active treatment. t-MNs have been categorized into an alkylating agent class and a topoisomerase II inhibitor class [1, 10]. However, patients are often exposed to multiagent combination CT or combined modality CT plus RT, making it difficult to clearly classify patients into one of the two categories [11]. It is known that the prognosis of t-MNs generally follows the cytogenetic risk category of de novo AML [11, 12]. However, a more refined classification according to their biologic features is required for the better risk stratification and improvement of OS.

Based on this background, we retrospectively evaluated treatment outcomes and prognostic factors in adult patients with non-APL t-MNs who received any disease-course-modifying active treatment, particularly focusing on the role of cytogenetics.

## Materials and methods

### Patients and ethics statement

Patients were included in our study if they were 1) diagnosed with MDS or AML according to the 2008 revision of the WHO classification at Seoul National University Hospital (SNUH) from January 2004 to May 2017, 2) aged ≥ 18 years at the time of diagnosis of MDS or AML, and 3) previously treated with CT/RT and/or radioiodine therapy for the treatment of PC. If the PC was diagnosed within 6 months before the diagnosis of MDS or AML, the patients were excluded considering the possibility of double primary cancers. Patients diagnosed with t-MNs other than t-MDS or t-AML, such as therapy-related myeloproliferative neoplasm (t-MPN) or t-MDS/MPN, were not included. Patients who received BSC with or without palliative cytoreduction only and patients with t-APL were excluded.

The present study was performed in accordance with the ethical principles in the Declaration of Helsinki and its later revision in 2013. This study was approved by the Institutional Review Board (IRB) of SNUH, Seoul, Korea (Approval Number: 1709-054-883). Informed consent was waived by the IRB, considering the retrospective nature of this investigation.

### Definitions and analyses of cytogenetics

Cytogenetic studies using standard G-banding techniques on heparinized bone marrow aspirate samples were performed as part of the diagnostic work-up. At least 20 metaphases were analyzed whenever possible Karyotypes were recorded according to the International System for Human Cytogenetic Nomenclature 2013. AML with t(8;21)(q22;q22), inv(16)(p13;q22), and t(16;16)(p13;q22) were considered core binding factor AML (CBF-AML). A monosomal karyotype (MK) and complex karyotype (CK) were defined according to the 2017 European Leukemia Net (ELN) genetic risk classification [13] as follows: Briefly, MK denotes the presence of ≥ 2 distinct autosomal chromosome monosomies or a single autosomal chromosome monosomy in combination with ≥ 1 structural chromosomal abnormalities, excluding CBF-AML. CK denotes ≥ 3 unrelated chromosomal abnormalities in the absence of WHO-designated recurrent cytogenetic abnormalities. Balanced translocation involving ≥ 2 chromosomes was defined as a single abnormality because it leads to one fusion protein. By contrast, unbalanced translocations were counted as two abnormalities. CBF-AMLs were regarded as having a single abnormality even if they had any other additional chromosomal abnormalities. We classified patients into three cytogenetic risk groups, based on the 2017 ELN genetic risk classification [13] but without consideration of molecular abnormality. Although the ELN risk classification was derived from AML but not MDS, we applied the cytogenetic risk classification to both t-MDS and t-AML, as a previous study [4], because t-MDS are often considered almost same as t-AML because of sharing etiology, particularly poor prognosis, and eventual progression to t-AML in a short time [14].

### Statistical analysis

OS was defined from the time of diagnosis of t-MN to death from any cause. In the comparison of OS according to risk groups, we used allogeneic HSCT-censored OS (AC-OS) because we intended to estimate the pure effect of cytogenetic aberrations regardless of the patient characteristics and treatment strategies. Survival was analyzed with the Kaplan-Meier method and compared by a log-rank test. A multivariate analysis was conducted by entering a backward Cox regression analysis, with variables of *P* < 0.1 in the univariate analysis. Fisher’s exact test or Pearson’s chi-square test was used as appropriate for determination of non-random associations between two categorical variables. Each value was two-sided with an accepted level of statistical significance at *P* < 0.05. All analyses were conducted with SPSS version 19.0.1.

## Results

### Selection of actively treated t-MN patients (N = 72)

**Fig 1:**
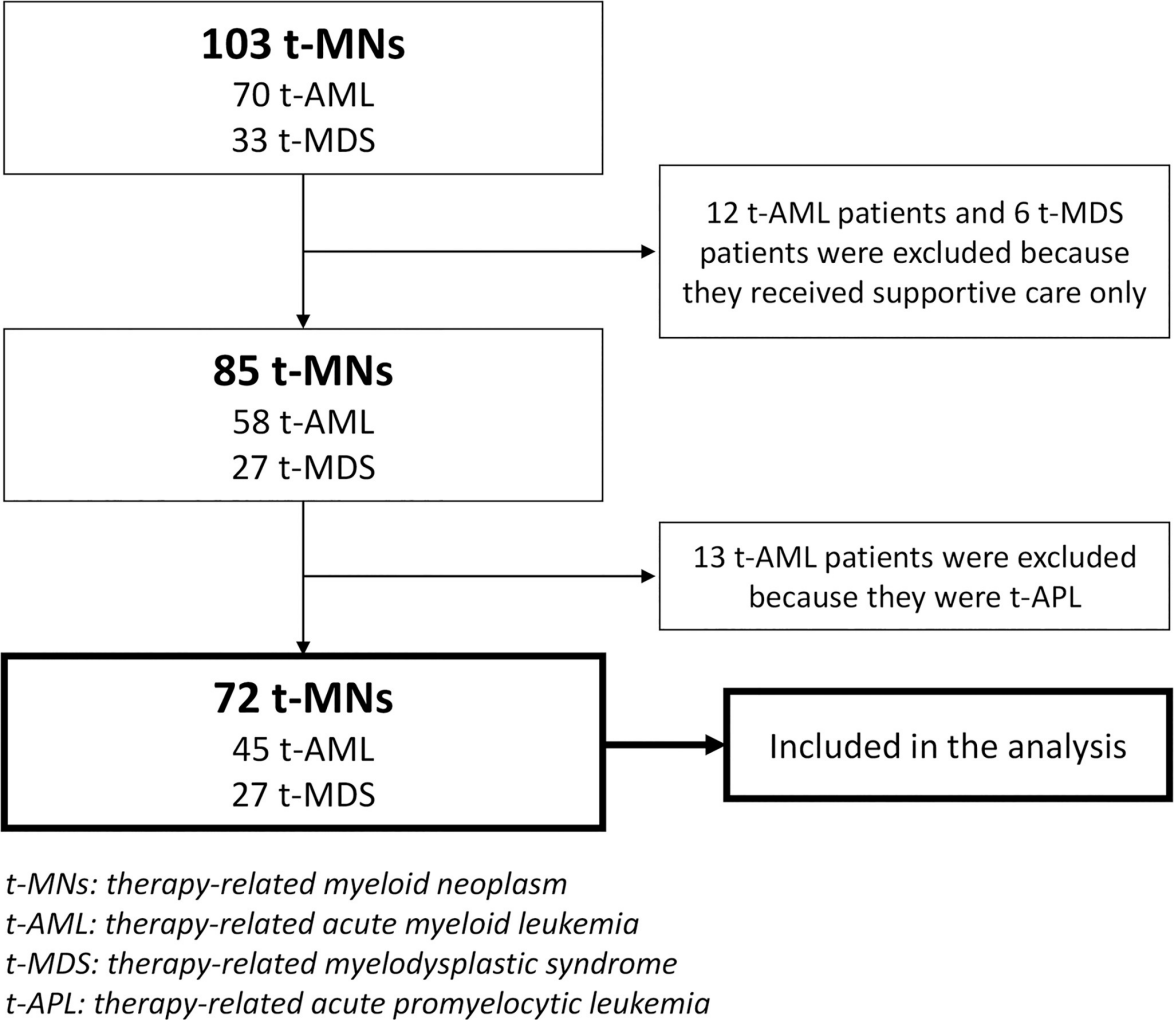
Selection of analyzed patients.

**Fig 2:**
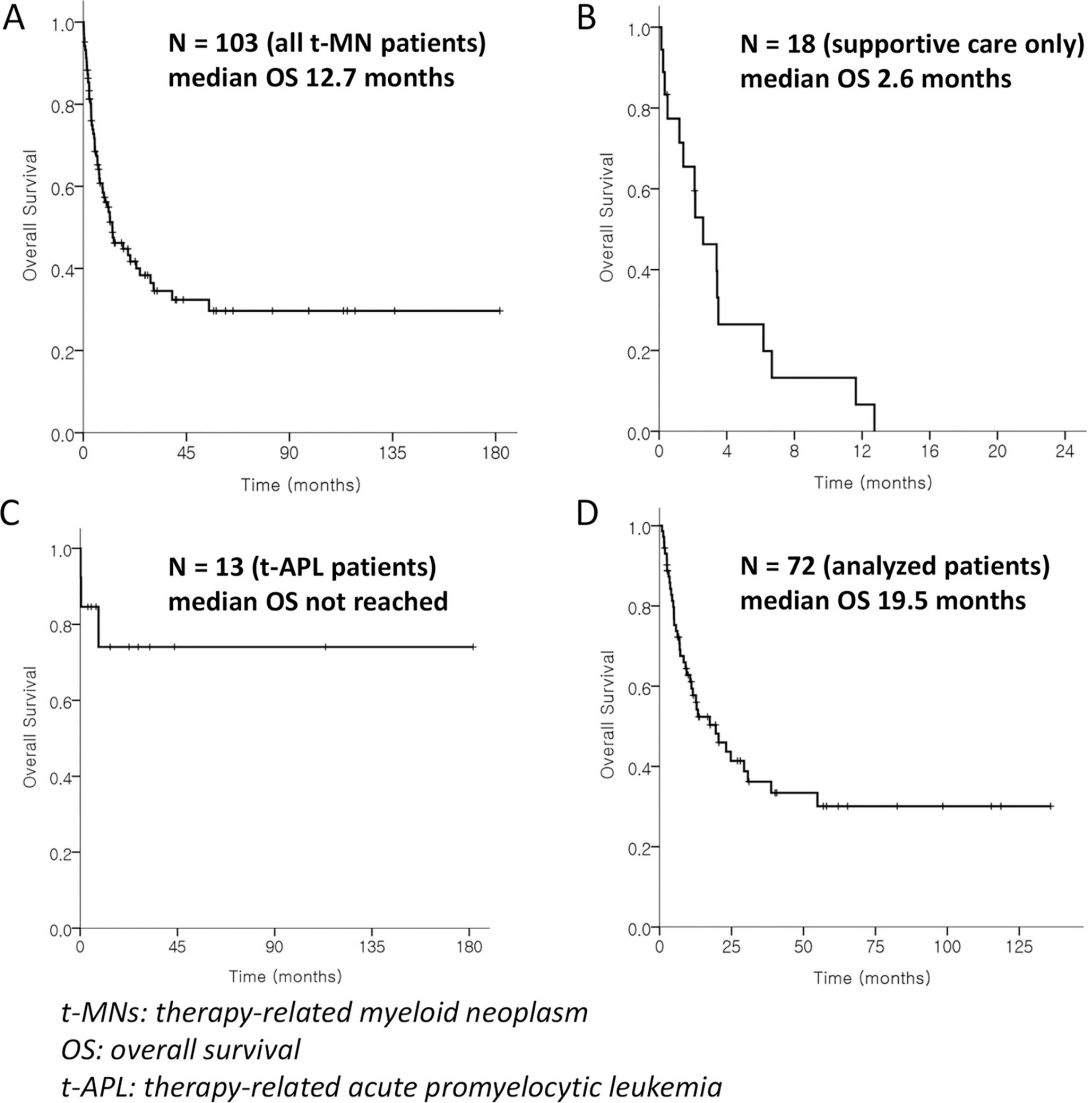
Kaplan-Meier curves of overall survival. (A) all patients with therapy-related myeloid neoplasms (N = 103); (B) patients who received supportive care only (N = 18); (C) patients diagnosed with therapy-related acute promyelocytic leukemia (N = 13); (D) the analyzed patients (N = 72).

With a median follow-up time of 31.1 months [95% confidence interval (CI) 17.2–45.0)], 103 consecutive patients with t-MNs were initially defined (Fig 1). Their median OS was 12.7 months (95% CI 5.4–20.0; Fig 2A). Eighteen patients received BSC only, with or without palliative cytoreduction with either hydroxyurea or low-dose cytarabine. Their median age at the time of t-MN diagnosis was 60 (range 20–82), and their median OS was 2.6 months (95% CI 1.0–4.2; Fig 2B). Thirteen patients were diagnosed with t-APL, and all of them received IC plus ATRA. Three of them experienced early death during induction IC, and their 3-year OS was 78% (Fig 2C). Therefore, 72 patients (45 with t-AML and 27 with t-MDS) were finally selected (Fig 1), and their median OS was 19.5 months (95% CI 8.8–30.3; Fig 2D).

### Patient characteristics

The key characteristics of the 72 patients are presented in Table 1. The median age at the diagnosis of PC and t-MNs was 51 years (range, 9–77) and 56 years (range, 19–82), respectively. Lymphoma and breast cancer were the dominant PCs associated with t-MNs; furthermore, 62.5% of the patients had been exposed to two or more kinds of CT and/or RT, either concurrently or sequentially. Only five patients had less than 20 metaphases of chromosome (18 metaphases in two patients, and 14, 6, and 5 metaphases of chromosome in one patient, respectively). Stratifying the patients according to the three cytogenetic risk categories, 10 patients (13.9%) showed favorable risk cytogenetics, i.e., CBF t-AML, 32 patients (44.4%) showed intermediate-risk cytogenetic features, and 30 patients (41.7%) showed adverse-risk cytogenetic features. Among 20 patients who had a normal diploid karyotype (NK), mutations of *FLT3*-ITD and *NPM1* were tested in 7 and 6 patients, respectively, and the results were all wild-type.

**Table 1:** Patient characteristics.

Parameters		Entire patients	NK	Non-NK intermediate risk	P-value (NK vs. non-NK int.)
Patients number		72	20	12	
Age at t-MN diagnosis	Median; years (range)	56 (19–82)	58 (21–77)	56 (23–77)	0.415
	≥ 60 years	31 (43.1%)	10 (50.0%)	5 (41.7%)	0.647
	≥ 70 years	11 (15.3%)	1 (5.0%)	3 (25.0%)	0.136
Age at PC diagnosis	Median, years (range)	53 (9–77)	50.5 (15–73)	54.5 (23–77)	0.328
Time from PC diagnosis to t-MNs diagnosis	Median; months (range)	32.7 (6.0–317.0)	38.1 (6.0–138.3)	24.1 (6.0–127.4)	0.519
Sex	Male	30 (41.7%)	7 (35%)	4 (33.3%)	1.000
Types of t-MN	t-MDS	27 (37.5%)	4 (20.0%)	8 (66.7%)	0.021
	t-AML	45 (62.5%)	16 (80.0%)	4 (33.3%)	
Types of PC	Lymphoma	21 (29.2%)	7	2	-
	Breast cancer	13 (18.1%)	4	3	
	Gastrointestinal cancers	9 (12.5%)	2	1	
	Gynecologic cancers	5 (6.9%)	1	2	
	Germ cell tumors	4 (5.6%)	1	1	
	Thyroid cancer	4 (5.6%)	2	0	
	Others	16	3	3	
PC treatment*	Alkylators	60 (83.3%)	15	10	-
	Topoisomerase II inhibitors	41 (56.9%)	12	7	
	Radiation therapy	25 (34.7%)	6	4	
	Radioactive iodine	4 (5.6%)	2	0	
	One of the above	27 (37.5%)	9	5	
	Two of the above	32 (44.4%)	7	5	
	Three of the above	13 (18.1%)	4	2	
Uncontrolled PC at t-MN diagnosis	Yes	17 (23.6%)	2 (10.0%)	2 (16.7%)	0.620
Cytogenetic risk category^†^	Favorable	10 (13.9%)			-
	Intermediate	32 (44.4%)		12 (100%)	
	Adverse	30 (41.7%)	20 (100%)		
Monosomal karyotype	Yes	18 (25.0%)	0	0	-
Complex karyotype	Yes	28 (38.9%)	0	0	-
NK	Yes	20 (27.8%)	20 (100%)	0	-

NK, normal karyotype; t-MNs, therapy-related myeloid neoplasms; PC, preceding cancer; t-MDS, therapy-related myelodysplastic syndrome; t-AML, therapy-related acute myeloid leukemia

*A patient may be exposed to two or more PC treatments

†category modified from the European LeukemiaNet 2017 classification (without consideration of molecular abnormality)

### Response rate of patients

Among 45 patients with t-AML, 43 patients received induction IC as front-line treatment and 24 patients (55.8%) achieved a CR after one or two courses of induction IC. The other 2 patients were initially treated with hypomethylating agents (HMAs); one achieved a CR, and the other exhibited partial remission with significant hematologic improvement (HI). The CR rates according to the cytogenetic risk groups were 66.6% (6 out of 9 patients), 70% (14 out of 20 patients), and 28.6% (4 out of 14 patients) for favorable, intermediate, and adverse risks, respectively. In the intermediate-risk group, 13 out of 16 patients with NK and 1 patient out of 4 patients with non-NK-intermediate-risk cytogenetics achieved a CR.

Among 27 patients with t-MDS, 6 (22.2%), 14 (51.9%), and 7 (25.9%) patients were classified into low-intermediate-, high-intermediate-, and high-risk categories, respectively, according to the International Prognostic Scoring System. Twenty-five patients were initially treated with HMAs, either azacitidine (N = 15) or decitabine (N = 10), and the other 2 patients received allogeneic HSCT directly (they spent 1.0 and 4.6 months respectively for donor selection with supportive cares and were censored at the time of allogeneic HSCT for AC-OS analysis). Among 25 patients who were treated with HMAs, 13 patients benefited from the therapy (1 patient with CR, 3 patients with a partial response, and 9 patients with any HI).

### Allogeneic HSCT-censored overall survival and overall survival according to the cytogenetic risk stratification

**Fig 3:**
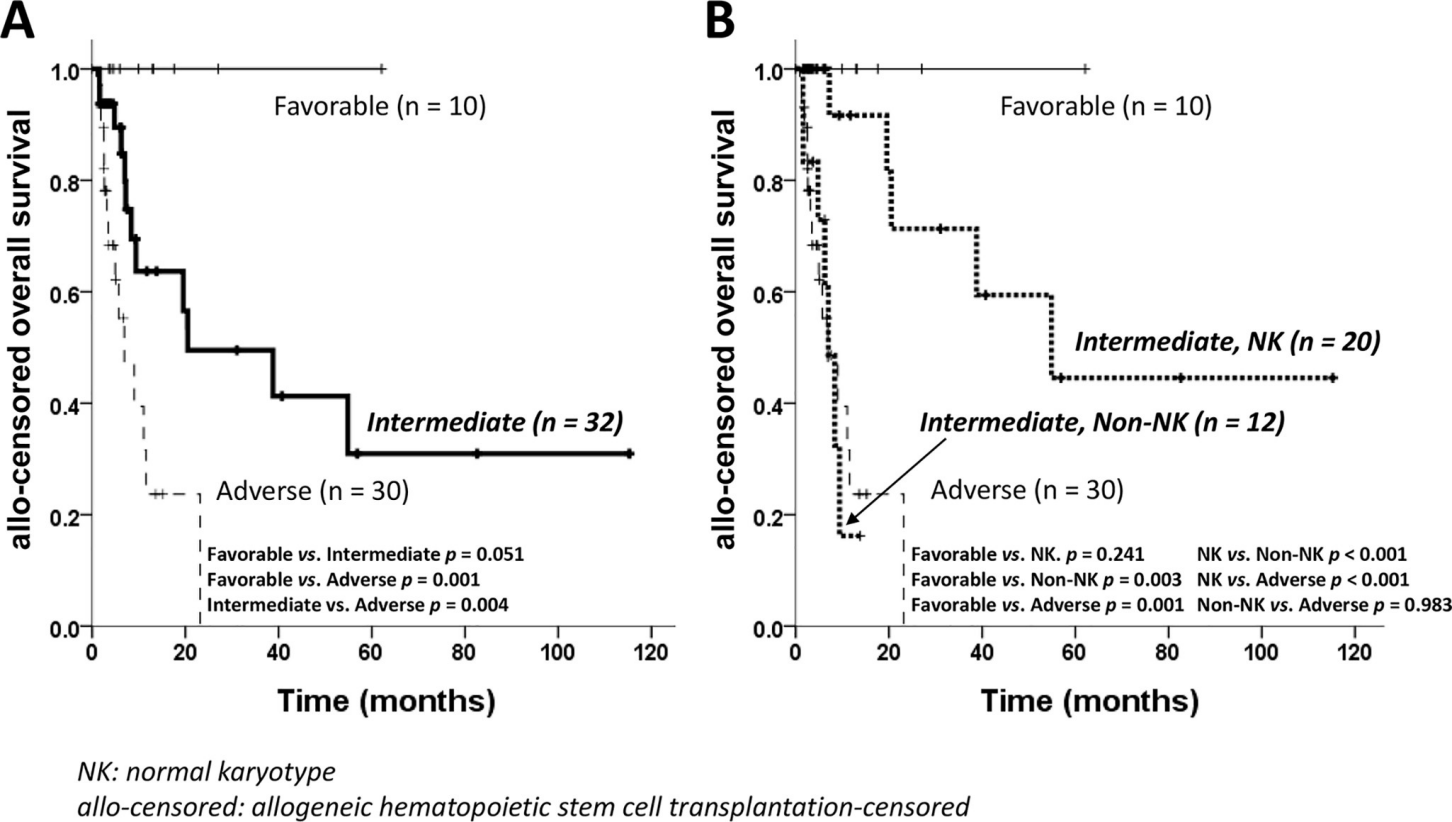
Allogeneic Hematopoietic stem cell-censored overall survival. (A) according to the cytogenetic risk category and (B) after the separation of normal karyotypes from non-normal karyotypes in the intermediate-risk group.

Twenty-five patients (34.7%) proceeded to allogeneic HSCT: 19 patients had t-AML, and 6 patients had t-MDS. The median AC-OS (not OS) of 72 patients was 20.5 months (95% CI, 8.10–32.90). The AC-OS curves were well separated according to the cytogenetic risk classification (Fig 3A). Of note, among 32 patients in the intermediate-risk group, 20 patients had NKs, whereas 12 patients had any cytogenetic abnormality. The median follow-up in the NK versus non-NK intermediate risk t-MN group was 56.9 months (95% CI, 22.2–91.6) and 13.8 months (95% CI, 9.6–18.0), respectively. A comparison of AC-OS between patients with NK vs. non-NK-intermediate-risk cytogenetics revealed that patients with NKs showed a superior AC-OS [median of 54.9 months (95% CI 17.6–92.2)] compared to those with non-NK-intermediate-risk cytogenetics [median of 7.0 months (95% CI, 4.5–9.5), *P* < 0.001; Fig 3B].

To investigate whether the AC-OS difference between two groups are a unique finding limited to patients with t-MNs, we compared AC-OS between patients with NK (N = 272) vs. patients with non-NK-intermediated risk cytogenetics (N = 112) from our actively treated, non-therapy-related AML cohort (N = 384). Unlike t-MNs patients, there was no significant difference of AC-OS between the two groups (5-year AC-OS 56.7% for patients with NK cytogenetics vs. 46.6% for patients with non-NK-intermediate cytogenetics, *P* = 0.149; S1 Fig).

**Fig 4:**
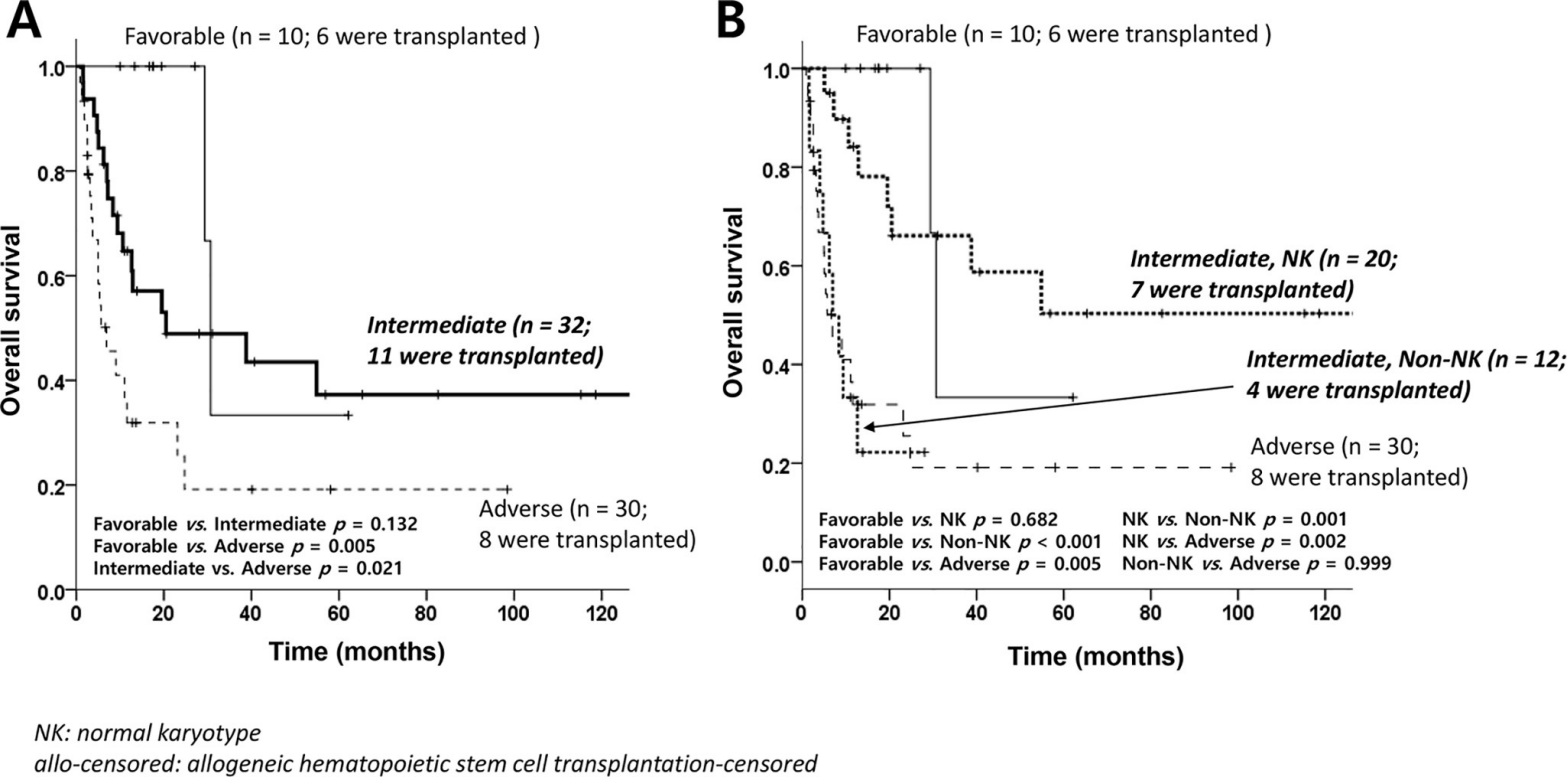
Overall survival. (A) according to the cytogenetic risk category and (B) after the separation of normal karyotypes from non-normal karyotypes in the intermediate-risk group.

We also evaluated OS analysis according to the cytogenetic risk. The significant difference between patients with NK and those with non-NK-intermediate cytogenetics were also observed (*P* = 0.001, Fig 4)

Because patients with non-NK intermediated cytogenetics showed inferior AC-OS compared to those with NK cytogenetics, we compared baseline patient characteristics between the two groups. Majority of patients with NK cytogenetics was t-AML (N = 16, 80.0%), whereas 8 out of 12 patients (66.7%) were t-MDS patients (P = 0.021 by Fisher’s exact test) in patients with non-NK intermediate risk cytogenetics. Otherwise, there was no difference of age, sex, and proportion of uncontrolled PC between the two groups (Table 1).

### Prognostic factors for allogeneic HSCT-censored overall survival in the analysis cohort

For the univariate and multivariate analyses for AC-OS, the cytogenetic risk category was modified by dichotomization of the favorable plus NK *vs*. the non-NK-intermediate plus adverse cytogenetic risk groups. In the univariate analysis, male sex, age ≥ 70 years old, t-MDS, non-NK-intermediate plus adverse cytogenetic risk, MK, and CK were associated with an inferior AC-OS. In the multivariate analysis, male sex, age ≥ 70 years old, and non-NK-intermediate plus adverse cytogenetic risk were remained as independent prognostic indicators for AC-OS (Table 2).

**Table 2:** Univariate and multivariate analysis for allogeneic hematopoietic stem cell transplantation-censored overall survival (N = 72).

Parameters		HR	95% CI	*P*
Univariate analysis				
Male vs. female		3.36	1.51–7.48	0.003
Age ≥ 40 vs. < 40 years		1.23	0.46–3.30	0.678
Age ≥ 50 vs. < 50 years		1.59	0.64–3.97	0.317
Age ≥ 60 vs. < 60 years		2.12	0.98–4.57	0.057
Age ≥ 70 vs. < 70 years		3.23	1.38–7.57	0.007
Uncontrolled preceding cancer (Yes vs. No)		1.23	0.52–2.92	0.64
t-MDS vs. t-AML		2.72	1.27–5.84	0.01
Non-NK-intermediate + adverse vs. Favorable + NK		15.05	4.19–54.07	< 0.001
Monosomal karyotype (Yes vs. No)		3.07	1.36–6.92	0.007
Complex karyotype (Yes vs. No)		3.36	1.49–7.61	0.004
Multivariate analysis				
Male vs. female		4.84	2.00–11.74	< 0.001
Age ≥ 70 vs. < 70		3.41	1.38–8.38	0.008
Non-NK-intermediate + adverse vs. Favorable + NK		24.13	5.73–101.60	< 0.001

HR, hazard ratio; CI, confidence interval; t-MDS, therapy-related myelodysplastic syndrome; t-AML, therapy-related acute myeloid leukemia; NK, normal karyotype

## Discussion

Our study showed that the cytogenetic risk category contributes to prognosis prediction, as previously reported [4, 12], and a more refined classification would be possible within intermediate-risk-group patients who underwent active treatment.

As for topoisomerase II inhibitor class t-MNs, DNA breaks caused by the drugs are known to frequently result in the direct induction of fusion oncogenes involving *RARα* at 17q21, *RUNX1* at 21q22, and *MLL* at 11q23 [7, 15], with a relatively shorter latency of 2~3 years [1]. Therefore, such drugs would mainly induce t-APL or CBF t-AML. By contrast, patients with alkylator class t-MNs often show an association with CK, MK, and abnormalities of chromosome 17, presented as a preceding period of t-MDS with a longer latency. In recent genomic studies of t-MNs using next-generation sequencing (NGS), the selection of pre-existing cell clones by cytotoxic therapy is gaining more support as being involved in the pathogenesis of alkylator class t-MNs than the existing theory of genome instability induced by cytotoxic therapy [1, 16]. Although information for the two categories of t-MNs is known, in many cases it is currently unclear how to predict the pathogenesis of t-MNs harboring NKs or other cytogenetic abnormalities mostly classified as intermediate risk. Moreover, because the current definition of t-MNs is just medical-history oriented, some of them may even have a possibility of being actually ‘therapy-after’ but not ‘therapy-related’ MNs. In this regard, more detailed analyses of the characteristics of t-MN patients who have intermediate-risk cytogenetics, including NKs, are important.

In our study, t-MDS patients had inferior AC-OS than those with t-AML in univariate analysis although not in multivariate analysis (Table 2). In addition, 16 out of 20 patients with NK cytogenetics was t-AML. One may think that patients with t-MDS have superior outcomes compared to those with t-AML considering natural disease courses of myeloid malignancies. However, not the entire t-MNs patients but patients who were ‘actively treated’ were included in our analysis. A part of patients who were classified as t-AML by definition (*i*.*e*., by medical history) might actually be patients with biologically closer to de novo AML with better performance status, and this might be one of reasons for better prognosis of patients with NK in our study.

The relatively better AC-OS and OS in patients with NKs in our study might be explained by their underlying mutational characteristics. Ok et al. conducted a retrospective analysis of 108 consecutive patients with t-MDS or t-AML treated at the MD Anderson Cancer Center and reported a strong prognostic relevance of *TP53* mutation [3]. *TP53* mutation was associated with CK (*P* < 0.0001), and patients who had *TP53* mutations showed an inferior OS compared to those with wild-type *TP53* (6.1 vs. 14.1 months; *P* < 0.0001). In the multivariate analysis, *TP53* mutation was an independent prognostic biomarker, whereas CK was not [3]. Interestingly, among the 16 patients who had NK, none of them harbored mutated *TP53* [3], suggesting that infrequent *TP53* mutation in patients with NK t-MNs may contribute to better outcomes. The result is in line with the result from a study from the Dana–Farber Cancer Institute regarding the genetic ontogeny-based classification of AML [17]. The researchers defined 3 groups of ontogeny-defining mutations, namely, the secondary-type, *TP53-*mutated, and de novo/pan-AML groups. When they evaluated 101 patients with t-AML among patients enrolled in a clinical trial, t-AML included all three genetic groups, and only 2 out of 20 patients with NKs harbored a *TP53* mutation [17].

Inferior survival outcomes in male compared to female patients with t-MNs have been reported in several studies in various settings: among t-MN patients who underwent IC [18] and with respect to the AC-OS of patients with t-MNs [3] and the OS of t-MN patients who received allogeneic HSCT [19]. t-MNs are generally known to have a female predominance [18, 20, 21], probably affected by the higher frequency of t-MNs after breast cancer. The potential prognostic relevance of sex in t-MN patients might be affected by either differences in the distribution of PCs or biological distinctions according to sex. This possibility needs further investigation.

In our study, most patients with t-AML and t-MDS received first-line therapy with induction IC and HMAs, respectively; the CR rate of induction IC in t-AML patients was 55.8%, and 55.6% of t-MN patients (15 out of 27 patients; 13 out of 25 with t-MDS and 2 out of 2 with t-AML) treated with HMAs achieved at least a HI. In earlier studies, the CR rates after induction IC in patients with t-MNs were reported to be low and short-lived, ranging from 27 to 37% [5, 6, 22]. However, recent studies show that the response rates are not so inferior, even reaching above 60% [11, 23]. Both the improvement of supportive care over decades and selection bias from the analyzed patient populations could be reasons for the difference. Currently, for better outcomes, it seems to be more important to create a tailored strategy for induction treatment according to genetic risk. In patients with CBF t-AML, studies report that the CR rates after induction IC are comparable to those in patients with de novo CBF-AML [24, 25], although the OS is inferior [11, 24–26] and is probably affected by older age [24, 25]. Lindsley et al. reported that the proportion of patients with secondary-type or *TP53*-mutated t-AML requiring two or more rounds of induction IC to achieve a CR was higher than the corresponding proportion of patients in the pan/de novo AML group. Considering the results, the development of better induction therapy is particularly challenging in patients with poor genetic profiles. The use of HMAs is an attractive approach because HMAs are effective in patients with secondary AML or with CK [27] and are possibly effective in patients with MNs harboring a *TP53* mutation [28]. HMAs are also advantageous because of their lower toxicity than intensive IC, considering that secondary AML populations are older [17]. Future studies applying either induction IC or HMAs according to cytogenetic or genetic profiles rather than the manifestation of t-MNs (i.e., t-MDS vs. t-AML) can be helpful to define better induction therapy.

Mutational data on the patients could not be included in the analyses of our study. The number of patients who had information on the mutational status of *FLT3* or *NPM* was insufficient to conduct analyses. Although it is known that t-MNs have significantly lower frequencies of *FLT3* and *NPM1* mutations than de novo MNs [1, 14], such mutations may have an impact on the prognosis in some t-MN patients, especially those with NKs. Information on *TP53* and other mutations estimated by the NGS test were conducted in only a few recently diagnosed patients, which is a major limitation of the present study. Since the characteristics of MDS and AML according to genetic features have been elucidated in detail, the NGS test, if available, should be integrated into the management of t-MN patients. However, cytogenetics will continue to play an important role. Poor cytogenetics such as CKs or MKs can be a surrogate of poor genetic features, and whether performing an NGS test in these populations is necessary and cost-effective is debatable. In addition, under personal or social situations where an NGS study is not available, planning of treatment and prediction of the prognosis according to cytogenetics will still be needed.

In conclusion, in patients with actively treated non-APL t-MNs, cytogenetics is a strong prognostic indicator of AC-OS, along with male sex and age ≥ 70 years. The superior AC-OS and OS of patients with t-MNs who had NKs compared to those with non-NK-intermediate-risk cytogenetics suggests that a more-refined subdivision of risk stratification in t-MN patients with intermediate-risk cytogenetics is required.

## Supporting information

S1 Fig Difference of allo-censored overall survival between actively treated patients with normal karyotype (NK) vs. those with non-NK-intermediate risk cytogenetics in non-therapy-related acute myeloid leukemia cohort (N = 384). (PDF)

S1 Table Anonymized minimal data set of the analyzed patients (xlsx). (XLSX)
