# Supplementary material for: Better treatment outcomes in patients with actively treated therapy-related myeloid neoplasms harboring a normal karyotype
Source: PLoS One. 2018 Dec 31;13(12):e0209800. doi: 10.1371/journal.pone.0209800 (PMC6312245; doi:10.1371/journal.pone.0209800)
Supplement: S1 Fig — (PDF) [file pone.0209800.s001.pdf]

Supporting Information

S1 .Fig Difference of allo-censored overall survival between actively treated patients with normal karyotype (NK) vs. those with non-NK-intermediate risk cytogenetics in our non-therapy-related acute myeloid leukemia cohort (N = 384)

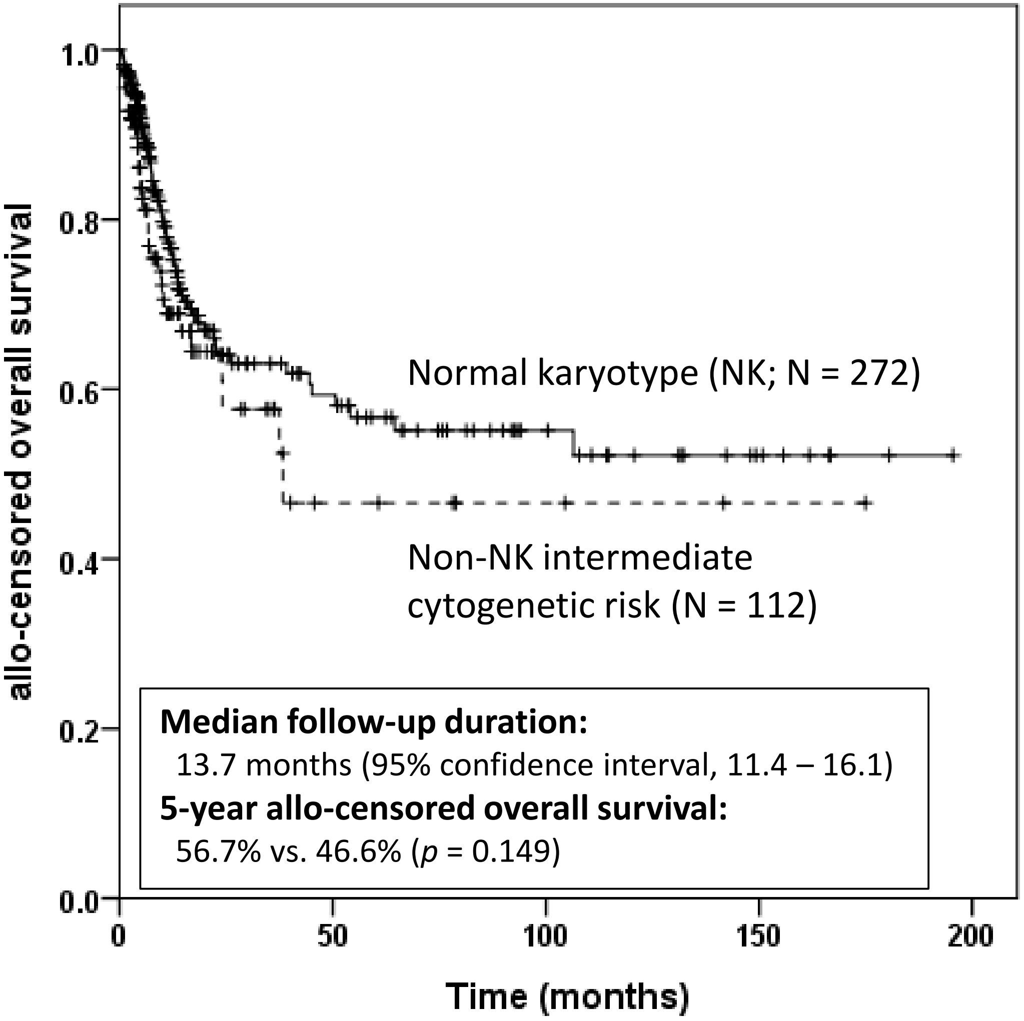

allo-censored: allogeneic hematopoietic stem cell transplantation-censored
